# How to Develop an Online Video for Teaching Health Procedural Skills: Tutorial for Health Educators New to Video Production

**DOI:** 10.2196/51740

**Published:** 2024-08-07

**Authors:** Komal Srinivasa, Amanda Charlton, Fiona Moir, Felicity Goodyear-Smith

**Affiliations:** 1 Department of General Practice & Primary Health Care The University of Auckland Auckland New Zealand; 2 Department of Histopathology Auckland City Hospital Auckland New Zealand; 3 Department of Molecular Medicine and Pathology The University of Auckland Auckland New Zealand

**Keywords:** online video, developing video, procedural video, medical education, clinician educator, health education

## Abstract

**Background:**

Clinician educators are experts in procedural skills that students need to learn. Some clinician educators are interested in creating their own procedural videos but are typically not experts in video production, and there is limited information on this topic in the clinical education literature. Therefore, we present a tutorial for clinician educators to develop a procedural video.

**Objective:**

We describe the steps needed to develop a medical procedural video from the perspective of a clinician educator new to creating videos, informed by best practices as evidenced by the literature. We also produce a checklist of elements that ensure a quality video. Finally, we identify the barriers and facilitators to making such a video.

**Methods:**

We used the example of processing a piece of skeletal muscle in a pathology laboratory to make a video. We developed the video by dividing it into 3 phases: preproduction, production, and postproduction. After writing the learning outcomes, we created a storyboard and script, which were validated by subject matter and audiovisual experts**.** Photos and videos were captured on a digital camera mounted on a monopod. Video editing software was used to sequence the video clips and photos, insert text and audio narration, and generate closed captions. The finished video was uploaded to YouTube (Google) and then inserted into open-source authoring software to enable an interactive quiz.

**Results:**

The final video was 4 minutes and 4 seconds long and took 70 hours to create. The final video included audio narration, closed captioning, bookmarks, and an interactive quiz. We identified that an effective video has six key factors: (1) clear learning outcomes, (2) being engaging, (3) being learner-centric, (4) incorporating principles of multimedia learning, (5) incorporating adult learning theories, and (6) being of high audiovisual quality. To ensure educational quality, we developed a checklist of elements that educators can use to develop a video. One of the barriers to creating procedural videos for a clinician educator who is new to making videos is the significant time commitment to build videography and editing skills. The facilitators for developing an online video include creating a community of practice and repeated skill-building rehearsals using simulations.

**Conclusions:**

We outlined the steps in procedural video production and developed a checklist of quality elements. These steps and the checklist can guide a clinician educator in creating a quality video while recognizing the time, technical, and cognitive requirements*.*

## Introduction

Up to 87% of surgical trainees routinely watch online laparoscopic videos as a part of multimedia learning, meeting a “user demand” [1]. Research has demonstrated that users consider online videos significantly more helpful than other resources due to improved self-confidence and navigability of the resource [2]. Furthermore, videos enhance active learning components for health professionals, foster a community of inquiry, and facilitate social interactions in online courses [3].

From an educator’s perspective, there are pedagogical reasons for using videos, such as creating a student-centered learning environment by enabling students to take an active role in their learning, increasing student engagement, and demonstrating procedures in a standardized and stepwise fashion [4]. While videos can be educational and interactive, these qualities depend on how they are designed and used in a learning context.

There are many options for health educators wanting to create a procedural video, ranging from outsourcing the entire process to doing it all themselves. Educators can engage professionals from the media production department in their institutions or private companies. They can also collaborate with students, residents, and colleagues with experience and interest in creating videos. The choice depends on the time, skills, funding, and, most importantly, the level of involvement the educator desires.

While the reasons for using an online educational video are numerous and well-researched, there is limited literature on developing a high-quality medical educational online video for teaching procedural skills. Several studies have shown that 10%-40% of medical videos on YouTube lack essential safety information [5,6]. Several studies have assessed the quality and content of medical procedural videos on YouTube and found them to be of variable educational value [7,8]. Health educators aiming to create high-quality videos will benefit from a clear understanding of the video development process and the required production skills. Therefore, we provide stepwise guidance and a quality checklist and identify barriers and facilitators to make a quality procedural video.

In this tutorial, we present the adaption of a previously described structure of dividing the development of a video into (1) preproduction, (2) production, and (3) postproduction phases to create a medical procedural educational online video. These phases are further divided into background factors and practical applications.

## Methods

### Preproduction Phase

#### Background Factors in the Preproduction Phase

A scoping review before starting the video recording process and previous attendance at courses on clinical education theories informed the steps of the process. In the preproduction phase, certain background factors need to be considered which are listed below in detail.

#### Needs Analysis

A video’s purpose, format, and content can be determined by performing a needs assessment before incorporating the video into a lesson plan [9].

#### Learning Outcomes

The learning outcomes should be clearly defined and aligned with an assessment taxonomy for clinical skills, such as Miller’s pyramid [10], as a video relates to procedural skills acquisition. Miller’s pyramid is a clinical assessment framework that defines a learner’s ability into the knows, knows how, shows how, and does categories that test progress from basic knowledge to practical application [10].

#### Learner-Centered

Allowing for different learning preferences will ensure maximal student engagement and active learning and contribute to a learner-centered environment [4].

#### Mayer’s Principles of Multimedia Learning

Different parts of the cerebral cortex process audio and visual content, and only a certain amount of information is held in working memory at any given time (cognitive load). Applying Mayer’s principles of multimedia while developing e-learning resources can reduce cognitive overload [11] as these systems can become overwhelmed [12]. These include 12 principles, such as signaling (visual cues are added to multimedia to signal the main concepts), coherence (unnecessary content is removed), and segmentation (an online video is broken into small units or pieces to enable the learner to process this information before moving to the next stage) [11,12]. While narration (using the educator’s voice) is essential [12], adding other audio media, such as music or background noise, adds to the cognitive load [3]. Multimedia principles should be considered when weighing up the optimal combination of text, images, and narration to communicate information.

#### Guidelines for the Design of Instructional Videos

In 2022, Meij and Hopfner suggested guidelines for the design of instructional videos for software design, which can also provide guidance for medical instructional videos [13]. These include (1) keeping instructional videos short, (2) supporting users in finding a suitable video by including the video purpose in the title, (3) previewing the task, (4) using a screencast with narration, (5) supporting an action-oriented approach, (6) consider key components of a well-designed procedure, (7) make task demonstration easy to follow and mimic, (8) support users in handling the transitory nature of the video, (9) review the task, (10) strengthen demonstration with practice, and (11) occasionally include music.

In addition to these, a clinical educator should be aware that the current video size guidelines for full high-definition videos are 1920×1080 pixels and 149 MB per minute [14].

#### Storyboard, Script, and Shot List

Preparing a storyboard, script, and shot list outlining the exact steps and scenes of the video optimizes the chance of smooth recording on the day [15,16]. If the content is unfamiliar then this should be peer-reviewed by a subject matter expert (who confirms the subject content is accurate and in line with the learning outcomes). For advice on audiovisual matters, checking the storyboard and shot list with an audiovisual expert would be useful [9,15]. The audiovisual expert is a person trained in media production who has expertise in producing videos. They may be from the audiovisual department of the organization or an outside company. Following this process ensures optimal video quality [15]. Validating the video script may address 6 questions related to the video’s objective, content, relevance, environment, verbal language, and inclusion of topics [15]. A knowledge of common video recording language and techniques will help inform the writing of the storyboard and shot list [12]. These include (1) framing a shot, (2) camera placement, (3) camera angle, (4) zooming in or out, (5) panning, and (6) cutting with purpose. These are explained further in Table 1.

**Table 1:** Common video recording language and techniques.

Technical word	Definition
Framing a shot	A shot is what is presented on screen. So, aim for the smallest frame necessary for the shot to balance detail and context.
Camera placement	The placement of the camera is essential. Examples include the 180-degree rule and the 30-degree rule. The 180-degree rule states that when recording 2 objects, the camera placement should not exceed 180 degrees from each other to provide consistency. The 30-degree rule states that the camera angles should be at least 30 degrees apart when changing angles.
Camera angle	Camera angles refer to the placement of the camera (ie, what the viewer sees). Examples include eye level, low angle, high angle, and bird’s eye, each with a different purpose. Tilting up or down is changing camera angles while the video is still rolling.
Zooming	It adjusts the focal length of the lens (in or out).
Panning	It is moving the camera from left to right.
Cutting with purpose	Is moving between scenes or camera angles on purpose.

#### Video Duration

The duration of an online video is another factor to be considered. There is no consensus on the optimal length of a procedural video, as this depends on several contextual factors. Videos of shorter duration have been shown to have better viewer engagement, and some authors state that videos should be limited to 6 minutes or less [4,17]. Others suggest they should be 10-15 minutes long [3]. Ideally, the length should range from 6 to 15 minutes, and videos longer than this should be subdivided into shorter parts or chapters labeled with time-stamped content [3,4].

#### Title

A video title should make the learner aware of the video’s goal and the session’s intended learning outcomes [13]. A good title increases the ease with which an educator or learner can search for the online video [13].

#### Educator Presence and Narration

Having the educator’s face or head and shoulders in the video and narrating with the educator’s voice [18] is also suggested to increase the learner’s connectedness with the content and educator [3]. This is especially true in asynchronous and remote learning situations where this “human touch” improves a learner’s sense of teacher presence. This improved sense of connectivity, either in a planned lesson or during self-directed learning, can lead to a more learner-centered environment, promote active learning, and create a sense of community [18]. Audio narration in a conversational and enthusiastic tone (Mayer’s principle of personalization) and at a reasonably fast pace (185-254 words per minute) is preferable [17].

#### Table of Contents and Time Stamping

A table of contents with links to specific time points (time stamping, chapters, or bookmarks) within a video also increases user navigation and control. These features save time as well as improve the ease of access and functional interactivity [17]. In instructional videos, interactive features such as pauses and quizzes can test the learners’ knowledge, enable reflection, and improve engagement [17].

#### Health Information Governance

Health information governance issues are essential in the preproduction phase and can be divided into ethical or professional considerations. Ethical approval might be required from specific jurisdictions and institutional groups before recording. Any patient images (still or video form) must be collected after their consent (in some jurisdictions), used respectfully, and stored to ensure patient privacy and confidentiality. Professional considerations relate to copyright, data protection, and indigenous populations’ sovereignty issues. As using content created by someone else may have copyright issues, specific permission may be required, especially if the video content is modified. Live streaming procedures in surgical broadcasting or coaching require knowledge of confidentiality and health information laws [19]. The final video and raw data must be stored with data protection considerations. There may be Indigenous Populations’ data sovereignty issues to consider, such as Māori data sovereignty in New Zealand (Te Mana Raraunga Maori Data Sovereignty Network), the US Indigenous Data Sovereignty Network, and the International Indigenous Data Sovereignty Interest Group. Specific bodies within or outside a university or health care organization can provide guidelines on appropriate processes to follow for data sharing for teaching.

#### Time, Cost, Feasibility, and Permissions

Finally, before recording can commence, the project’s time, costs, and feasibility must be considered. Permission might be required before recording at sites such as laboratories, hospitals, and university campuses. The cost and feasibility include a list of the necessary equipment for recording, the availability and the ability to use the equipment, and the cost of the entire project with a budget. The educator must consider the feasibility of recording time-dependent or rare procedures.

#### Making Our Video: Practical Considerations in the Preproduction Phase

**Figure 1:**
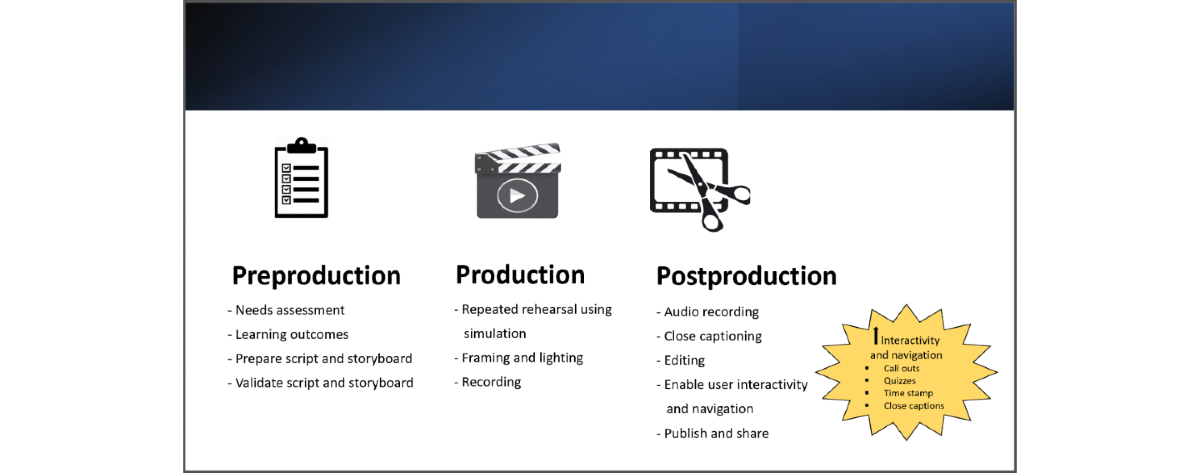
Steps in developing an online instructional video.

If doing the recording yourself, this stage should also involve attending courses on videography and editing skills and audiovisual skill building by deliberate practice with feedback, using simulations and rehearsals. Our steps for making an online video are shown in Figure 1.

We identified the target audience for this educational video (postgraduate pathology trainees and histology scientists), conducted a needs assessment, and listed the learning objectives from an educator’s perspective. We then converted these to learning outcomes from the learner’s perspective. We used the recommendations by Fleming et al [16] to guide our video’s script and storyboard, which also involved deciding the sequence of what, how, and where the recording would occur. The latter was structured in a table with 4-column labeled steps of the procedure, image or scene, audio (script), and photos (Multimedia Appendix 1).

A subject matter expert (AC), an anatomical pathologist with expertise in skeletal muscle pathology, assessed and validated the educational content of the storyboard and script. The technical content was evaluated by a media production contact from the Media Productions Department at the University of Auckland, who provided input on the technical aspects of the storyboard (Multimedia Appendix 2). We amended the storyboard and script to incorporate our experts’ suggestions. The final script and storyboard used for recording are presented in Multimedia Appendix 3. We created a checklist of the technical audiovisual equipment required on the day, listed under the production phase. We also needed to co-ordinate the participants’ schedules involved in the recording. Throughout this process, KS kept a journal to document the time each phase took. The repetitive cycle of feedback from the team and personal reflection led to the documentation of factors that had progressed well or not during each stage.

### Production Phase

#### Background Factors in the Production Phase

The background factors to consider in the production phase include ensuring all the equipment on the list is available, the videographer is familiar with using them, the audiovisual technical aspects of the recording process, and the planned recording schedule.

Ensuring that all equipment on the list is available and that the videographer is familiar with using them. The list consists of (1) adequate lighting, (2) a digital camera with a microphone, (3) a digital camera mount such as a monopod or tripod, (4) fully charged batteries, (5) a secure digital (SD) card with speed and capacity to record video, and (6) a method to transfer the video files from the camera to the computer, such as a Wi-Fi enabled connection, an SD to USB card reader, or a camera to computer cable.

An option is to record the images and videos on hand-held devices, such as personal mobile phones. This can be a cost-effective and convenient option, especially since current devices have sufficient camera resolution to produce high-quality images and increased memory storage options. The user is also familiar with using this device. However, there are patient confidentiality and data protection issues to consider before choosing to use a personal mobile phone. The use of personal mobile devices and personal cloud storage can potentially lead to breaches of data protection and patient privacy [20,21]. The shared video content may be widely accessible, and sometimes not in the way the authors of the video intended, such as by family members on a shared cloud storage. Currently, there is no health information governance legislation that applies worldwide. However, institutions and countries have specific guidance or legislation that must be adhered to (such as HIPAA [Health Insurance Portability Accountability Act] in the United States or the GDPR [General Data Protection Regulation] from the European Union).

Also, the audiovisual technical aspects of the recording process and the planned recording schedule must be considered. Choosing the appropriate demonstrator(s) for the video and rehearsing the recording process will streamline recording [12]. Tasks and responsibilities can be clarified before the day of recording. Recording more takes and angles than required may create a more time-effective and smoother video [12]. Ideally, the video should have different shots with judicious camera movements.

#### Making Our Video: Practical Considerations in the Production Phase

##### Setting

The video was recorded in a histopathology laboratory that routinely processes human skeletal muscle samples, as the video content was on macroscopically handling a fresh skeletal muscle biopsy. The videographer (KS) recorded a pathologist (AC) handling and preparing a fresh skeletal muscle biopsy in the laboratory. FM, FG-S, and AC provided feedback on the video. FM and FG-S are not experts in this content, and their feedback was from a naïve learner perspective informed by the literature. AC, a subject matter expert, also reviewed the initial video and provided feedback on content and editing.

##### Data Collection

The video was recorded immediately upon arrival of the specimen in the laboratory. KS recorded still images and short video clips using a digital camera mounted on a monopod. The process of recording the video clips and still photos took 4 hours. The equipment checklist, script, and storyboard created in the preproduction phase guided the recording. KS ensured all aspects of recording a particular step were completed before moving to the next step. Some steps needed rerecording as the initial images or shots were technically suboptimal. The suboptimal time-sensitive shots (as a muscle biopsy cannot be delayed in the fixation process upon receipt into the laboratory) were rerecorded immediately. In contrast, images of equipment or steps that were not time-sensitive were rerecorded at the end. FM and FG-S reviewed the initial video and provided feedback. AC provided feedback on the modified version of the video. KS iteratively modified the video in response to this feedback.

### Postproduction Phase

#### Background Factors in the Postproduction Phase

The background factors to consider in the postproduction phase include video editing [9,15,16], video hosting platforms, video quality, and health information governance issues. Various video editing software (such as Microsoft Video Editor, Camtasia [TechSmith], Adobe Premier Pro, iMovie [Apple Inc], Wondershare Filmora [Wondershare Technology] and many others) range in price, complexity, and ease of use. Technical considerations include internet speeds and the video file type (.mp4 file type is preferred). The video editing should conform to the principles of multimedia learning [11] and add audio narration and optional subtitles (or close captioning) to improve accessibility and inclusivity. Educators can also add active learning to a video by embedding the video in software, such as H5P (H5P group), to create interactive videos with quizzes.

The video should be published on an online platform that provides easy access, for example, YouTube or embedded in the learner’s learning management system. Many online platforms include videos for both patient and medical personnel use, and the aim and content of these videos will differ as they target different audiences [22]. YouTube is open access and popular with users; however, in a study, only 12% of medical videos were from university channels and professional organizations [22]. Alternatively, professional organizations like The Royal College of Pathologists of Australasia can host videos on their website such as the open-access macroscopic cut-up manual videos. Novice learners are more likely to access online videos on YouTube.

On the other hand, faculty members are more likely to access videos on professional organization web pages or YouTube channels specific to the organization [23]. However, YouTube hosts videos of variable quality, and novice users may not be able to identify poor-quality ones [23]. This clearly could have safety implications, depending on the procedure being learned.

Finally, the video author must ensure that health information governance issues described in the preproduction phase are all fulfilled before the video is published online.

#### Making Our Video: Practical Considerations in the Postproduction Phase: Analysis

The analysis phase of making a video involves postproduction editing, refinement, and publishing on a platform. The recorded images were transferred from the SD card into a computer with sufficient hard drive capacity. Various software, each chosen for a different purpose, were used to edit the sequence of scenes into an online video. KS used Wondershare Filmora 11 software to edit the video clips due to familiarity with the software. The final .mp4 file was transferred to Panopto, so that close captions in English could be enabled. The authoring software H5P was used to add a quiz to the video (drag and drop boxes) to provide formative feedback to the students and make the video interactive. H5P use also allowed bookmarking within the video to improve user navigation. The factors that made an effective online video from an educational and technical point of view, derived from the scoping review and broader literature, were incorporated into the video during the editing stage. These factors are elaborated in the Discussion section.

### Ethical Considerations

We were granted ethical approval by the Auckland Health Research Ethics Committee (AHREC) on May 6, 2022 (ref AH23813). We obtained appropriate written patient consent before the recording. The patient data is anonymized, and no compensation was provided to the patient. The consent form and the online video will be stored according to the institutional ethics requirements.

## Results

### General Points About our Video

Our final video is 4 minutes and 4 seconds long. Its creation took 70 hours (20 hours of preproduction, 4 hours of recording, and 46 hours of editing and revising), which KS recorded manually in a journal. A link to the video is available at the following reference [24].

**Figure 2:**
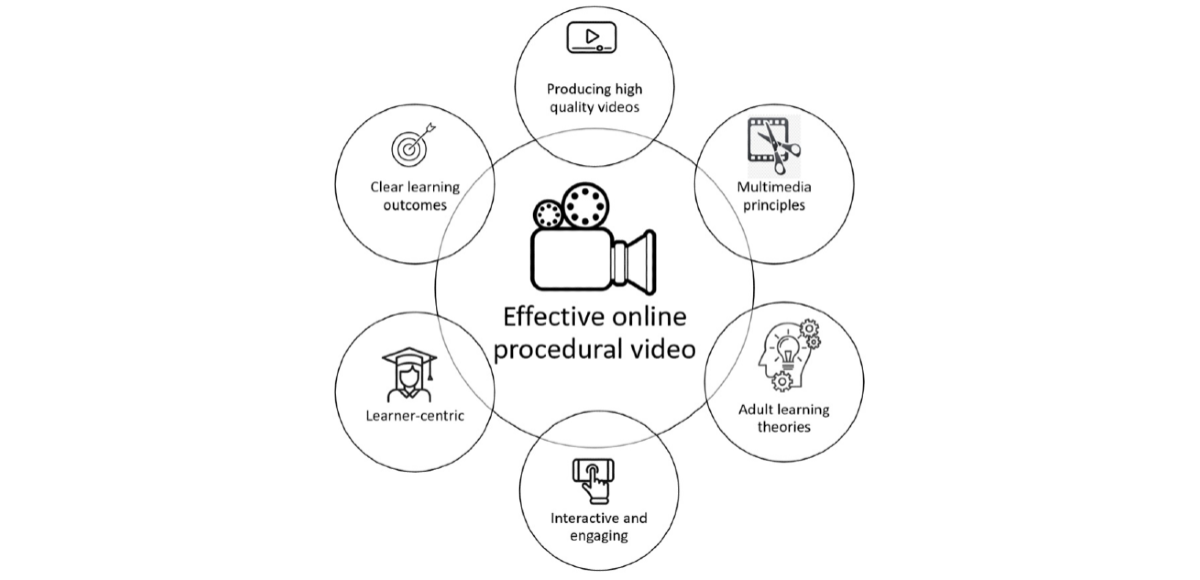
Diagrammatic depiction of components of an effective online procedural video.

We identified that a clinician educator creating an online educational video needs to align the video with clear learning outcomes and provide an engaging, learner-centric video that promotes active learning. Other aspects involve incorporating the principles of multimedia learning and adult learning theories and producing a video of high audiovisual technical quality. This is shown in diagrammatic form in Figure 2.

We also created a checklist of elements that ensures a high-educational quality video is produced based on previous literature on this topic and the process of developing a new video. These include a needs assessment, a validated storyboard and script, clear learning outcomes, a title that reflects video content, a video duration of less than 15 minutes, the face of the educator, narration by the educator, close captioning, and factors to enable interactivity with the video. The specific characteristics of our video that align with these elements are summarized in Table 2.

**Table 2:** Checklist of suggested quality elements in an online video.

Quality elements		Present in our video	Further details
Needs assessment		Not applicable	Needs assessment with learners will follow
Script and storyboard validated by an educational expert		Yes	—^a^
Script and storyboard validated by an audiovisual expert		Yes	—
Easy for a learner to access video		Yes	—
The title reflects the video content		Yes	—
Learning outcomes		Yes	—
Video duration <15 minutes		Yes	—
Time-stamping or bookmarks		Yes	—
Image of the instructor		Yes	—
Audio narration		Yes	—
**Principals of multimedia followed:**		Yes	—
	Signaling	—	Highlighting with arrows
	Coherence	—	Removal of extraneous material
	Personalization	—	The instructor’s voice was used for narration.
	Segmentation	—	Video is broken into small units or segments.
Functional interactivity		Yes	Interactive quiz inserted using H5P software
Close caption option		Yes	—

^a^Not applicable.

### Barriers and Facilitators to Creating Our Online Video

We documented the barriers and facilitators to creating an instructional video from the perspective of a clinician educator who was a novice at making videos of any type. One of the barriers to developing this video was the significant time required for creation. According to the literature, it takes about an hour to make 1 minute of online video content [25]. Still, with 70 hours of production time for a 4-minute video, the novice video producer (KS) vastly exceeded this. This time estimate is for experienced video creators or professionals. Due to inexperience, all phases of recording a video took a notable amount of time. The preproduction phase involved reading educational resources about theories of adult learning in the clinical setting and principles of multimedia and reading about the technical aspects of audiovisual recording [4,11-13,16,17]. The 46 hours of postproduction time included accessing and learning how to use the editing and interactive software, as well as revising the video based on iterative feedback.

The recording took 4 hours. Performing the videography also proved difficult, especially without previous rehearsal. For example, selecting camera settings, the way to frame a shot, and the lighting and camera placement to get an optimal picture had been learned in theory but proved challenging in practice. This was mainly due to KS having unfamiliarity with the equipment and a lack of technical videography skills. Several retakes were needed. It was also challenging to record shots to avoid confidential patient details. As this was not a simulated procedure, shots could not be repeated, and because the tissue was fresh, the procedure was time-critical.

A facilitator of video creation is the use of deliberate practice with feedback using simulation and rehearsals. The planned rehearsal using a simulated specimen could not be done, therefore technical skills on the day were minimal [26]. Rehearsals would have also identified the extra equipment and the storyboard or shot board adjustments. Therefore, the recording session took longer than scheduled due to necessary repetitions. The videographer (KS) could have completed online video editing courses, visited some video recording sessions with the university’s audiovisual department before recording, and practiced video recording and editing using nonmedical subjects, as these practical skills are more important than reading theory around this topic. These steps would have reduced the time spent recording and editing the video as more suitable images would have been captured on the day of recording.

Another facilitator was creating a community of practice (CoP) through discussions with colleagues with various skills and expertise. These discussions included specific technical equipment, audiovisual techniques such as camera angles, and the software to edit the raw recording. This dynamic group helped troubleshoot solutions to practical problems, which was especially important as KS chose software that was not industry standard for editing.

Other facilitators are organized and flexible, resulting in less stress during the recording process. Having a storyboard or script and an equipment list is essential. We found that it was not easy to stick to the storyboard, but the task would have been chaotic without it. Throughout this process, being cognitively and psychologically flexible to change makes the job easier and less frustrating.

## Discussion

### Principal Findings

While online educational videos are widely used in medical education, there is a lack of literature on creating a high-quality video. Our tutorial provides a step-by-step method for developing a quality medical video for clinician educators new to video creation.

Numerous studies have shown that the quality of videos on online platforms is heterogeneous [6-8,27]. Therefore, we propose a checklist to ensure that the educational factors in a video are optimized (Table 2). This checklist is a synthesis of the literature on this topic, so using it may ensure a high-quality video that is interactive and creates an engaging and learner-centric environment.

The time required to gain proficiency in making a procedural online video efficiently is a potential barrier. An educator new to making videos requires considerable time to produce new medical video content [25]. The professional body that makes online videos for the University of California San Francisco medical school estimates that 9-30 hours of an educator’s time is needed when developing such content, in addition to professional videography personnel time [28]. This time range includes both the time required to learn to create video content and use the technology. Often, for the educator, this is a time in addition to usual work hours. However, this can be a rewarding process, and the time requirements may be lessened by preparing and validating a script and storyboard, repeated rehearsals, especially using simulations to improve skills, creating a CoP, and intentionally developing and transferring video production skills gained in nonmedical settings. The time requirements will also lessen over time as a clinician educator becomes more experienced in video production. As an alternative to the clinician educator learning video production skills, they can engage and work with others with audiovisual expertise to do the production, reducing the time requirements.

The amount of knowledge and skills required to create a quality online video means that a novice may find this task daunting. However, KS found this process was aided by developing a CoP—a supportive and educational system known to be valued by teaching staff [29]. A CoP allows people to connect and learn about a particular topic, such as creating online videos. Our CoP group has variable amounts of experience in developing online medical videos of various types, creating a technically and emotionally supportive environment. Such groups work on the background theory of constructivism as they allow for social learning and mentorship [30]. The iterative process of seeking and providing feedback also improved video quality.

While several quality assessment tools have been developed in the last 4 years [31-34], an easy-to-use tool is desirable to assess the quality of a procedural educational video. Berrocal et al [31] have a one-page rubric for peer-reviewing microlectures. The instructional video quality checklist is another quality assessment tool, that uses a 26-item checklist assessing aspects of educational design, source reliability, multimedia principle adherence, and accessibility [32]. The LAP-VEGaS (LAParoscopic surgery Video Educational GuidelineS) video assessment tool examines 9 elements of a video [33], but it only applies to laparoscopic videos.

The authors of the LAP-VEGaS guidelines and LAP-VEGaS video assessment tool suggest using these during the preproduction and production phases to ensure a video has high educational value [33,34]. Several studies have subsequently used either the LAP-VEGaS guidelines or assessment tools to assess the quality of online medical videos. When de’Angelis et al [7] used the guidelines to review the quality of videos on appendectomy available on YouTube, 36% of the videos showed poor image quality, audio and written commentary were rarely present, and the overall conformity to the LAP-VEGaS guidelines was low. A similar result was found when videos on robotic-assisted laparoscopic pyeloplasty available on YouTube were assessed using the LAP-VEGaS guidelines [35]. This highlights the use of quality assessment tools or checklists for medical procedural videos.

Using online videos to teach procedural skills is no substitute for hands-on teaching. Karadas and O’Brien have demonstrated that repeatedly watching a video without physical practice can lead to an illusion of skill acquisition, where the learner assumes to have a greater skill level than they have [36]. Instead, online videos ought to be a part of the learning cycle for practical skills, perhaps based on an experiential learning cycle, so that the online video is followed by repeated practice exercises, such as simulation, adequate supervision, and a feedback cycle [37].

### Strengths and Limitations

A strength of this tutorial is the use of theoretical considerations in the preproduction, production, and postproduction phases of making a high-quality video to demonstrate a procedural skill. Furthermore, we illustrate this with our own experience of making such a video from the perspective of a clinician-educator new to making videos, therefore the relevance and usefulness of this tutorial to an experienced video maker will be limited. Furthermore, our checklist of elements for a procedural video that aligns with educational and audiovisual quality factors needs further validation before implementing its use.

### Conclusion

The process of creating procedural online videos is rewarding; however, it takes significant time and cognitive requirements, especially for clinician educators new to the process. Barriers include the time required for deliberate practice to gain competence in video production. Facilitators include technical skill building by deliberate practice with feedback, using simulations and rehearsals, and intentionally developing and transferring video production skills gained in nonmedical settings. For some, creating a CoP is supportive.

## Appendix

Multimedia Appendix 1 Subject matter expert’s input storyboard and script creating a video.

Multimedia Appendix 2 Technical expert input storyboard and script creating a video.

Multimedia Appendix 3 Final script and storyboard for an online medical video (modified after both experts input).

## Abbreviations

AHREC: Auckland Health Research Ethics Committee
CoP: community of practice
GDPR: General Data Protection Regulation
HIPAA: Health Insurance Portability Accountability Act
LAP-VEGaS: LAParoscopic surgery Video Educational GuidelineS
SD card: secure digital card
