# Supplementary material for: How to Develop an Online Video for Teaching Health Procedural Skills: Tutorial for Health Educators New to Video Production
Source: JMIR Med Educ. 2024 Aug 7;10:e51740. doi: 10.2196/51740 (PMC11339575; doi:10.2196/51740)
Supplement: Multimedia Appendix 2 [file mededu_v10i1e51740_app2.docx]

**Title: processing skeletal muscle biopsy in a Pathology laboratory**

Audience: Pathology registrars, consultants and surgical registrars (people who do the biopsy)

Learning objectives as applied to Bloom’s digital taxonomy:

1. Explain the basic equipment required for skeletal muscle biopsy processing (Blooms taxonomy verb remember)
2. Explain to a colleague the basic steps of skeletal muscle biopsy processing (Blooms taxonomy verb understand)
3. How is the process different from “usual” tissue processing in the laboratory and samples submitted? (Blooms taxonomy understand)
4. What are the critical or safety steps when performing this?
5. Organise the steps of a muscle biopsy processing in the correct order (Blooms taxonomy analyse)
6. Develop your own teaching video using a piece of meat (Blooms taxonomy create)

| **Step** | **Images/Scenes** | **Audio/description (music/script)** | **Photos** |
| --- | --- | --- | --- |
| Title | processing a skeletal muscle biopsy in a Pathology laboratory |  | ADHB, UoA logo |
| Learning objectives | List of learning objectives (20s) | Narration  In this video we will go over equipment and how to explain the process to a colleague.  We will show how this process differs from others, and what safety aspects you should consider.  Finally we will look at how to get organised and do you own video. | List of LO shown:   1. Explain the basic equipment required for skeletal muscle biopsy processing (Blooms taxonomy verb remember) 2. Explain to a colleague the basic steps of skeletal muscle biopsy processing (Blooms taxonomy verb understand) 3. How is the process different from “usual” tissue processing in the laboratory? (Blooms taxonomy understand) 4. What are the critical or safety steps when performing this? 5. Organise the steps of a muscle biopsy processing in the correct order (Blooms taxonomy analyse) 6. Develop your own teaching video using a piece of meat (Blooms taxonomy create) |
| Step 1 | Personal protective equipment | Narration: First of all put on your personal protective equipment.  This is an important safety step | Show animation of gown, gloves and goggles 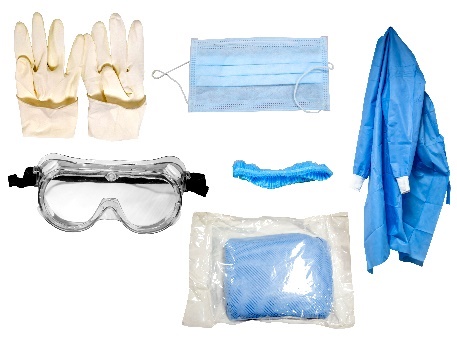  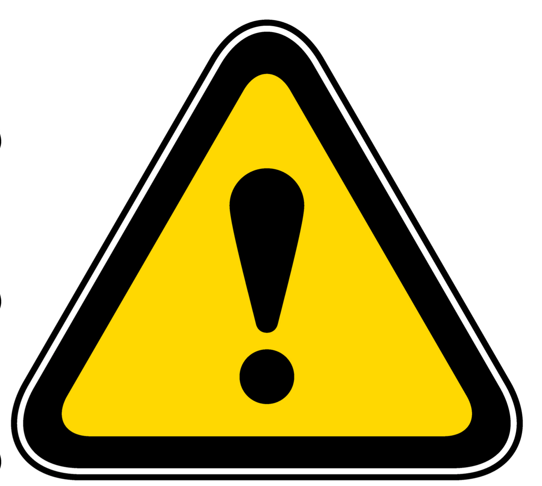 |
| Step 2 | Check patient details match form/specimen pot | Narration: Check that the patient details match those on the form and specimen pot | Scene showing actor checking/looking at form whilst wearing PPE |
| Step 3 | Check and prepare equipment  List equipment with picture of each appearing matching the voice over  Scalp blade to cut, petri dish to put muscle on, ruler, medium for EM, containers for freezing, cork for freezing, liquid nitrogen, isopentane | Narration: check and prepare the equipment | 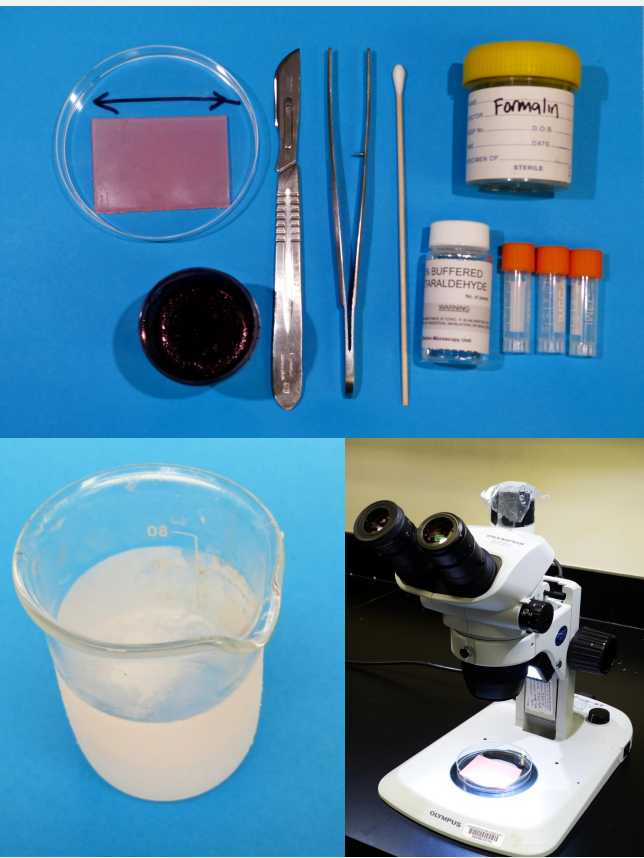 |
| Step 4 | Describe and measure muscle | Narration: The specimen is a red piece, _x _ x _mm in size | Actor measuring muscle |
| Step 5 | A Cut muscle and triage to different samples | Orientate the muscle using a dissecting microscope, cut and submit samples for mitochondrial studies, DNA storage, electron microscopy, snap frozen for histology, remainder in formalin for histology | Animation of the different samples to submit for 15 seconds  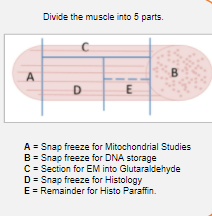 |
| Step 5 | B snap freeze muscle | The cut muscle is placed in moulding medium and snap frozen using liquid nitrogen. | Show actor cutting muscle and putting in different media etc  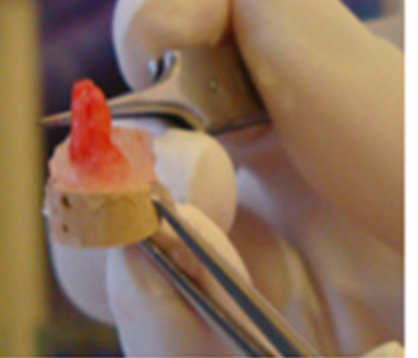 |
|  | C Place remainder of muscle in formalin | Orientate longitudinal and cross sections by using indian ink |  |
| Step 6 | Dispose of sharps safely | Make sure to dispose of the scalpel blade safely. | 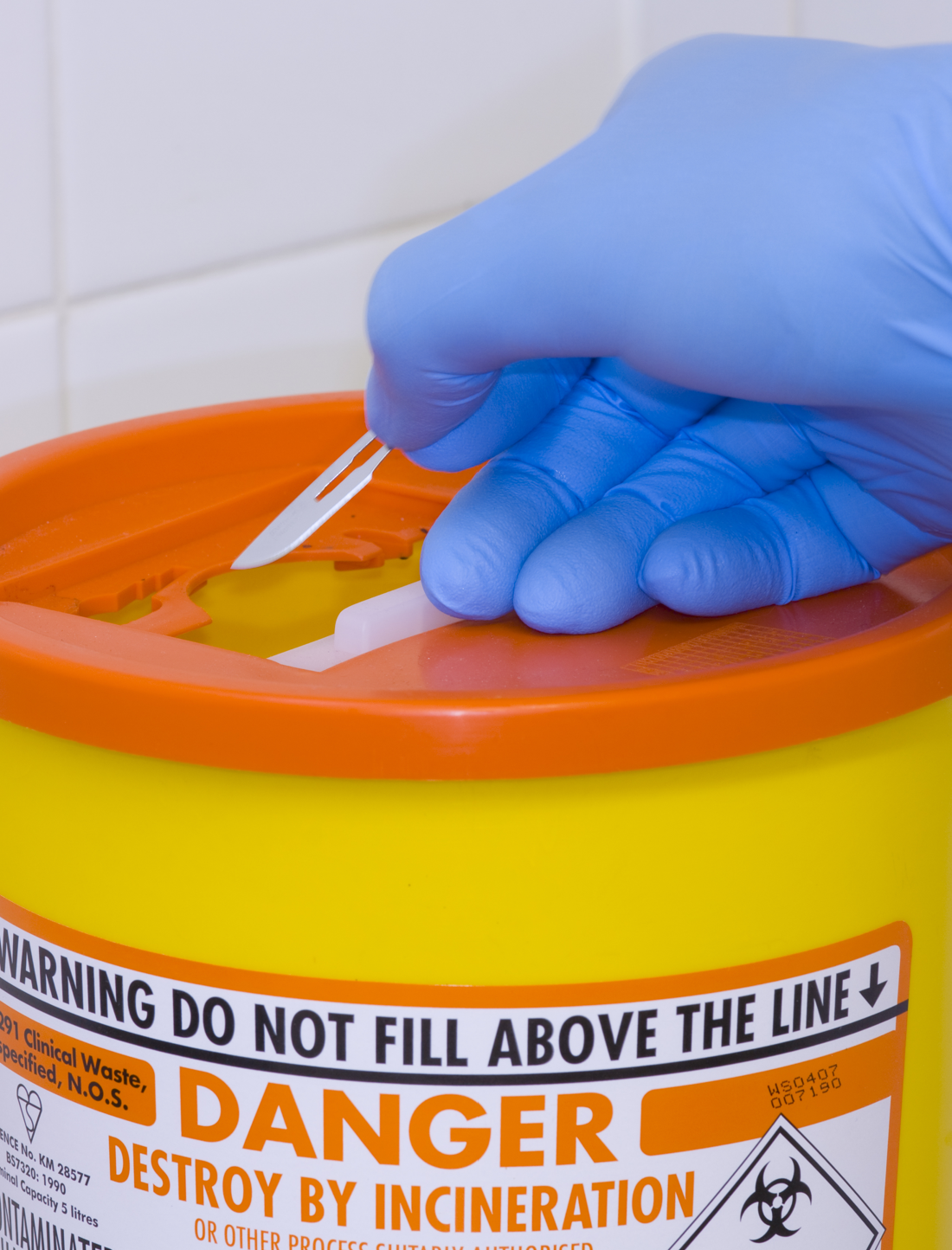 |
|  | Active learning:  Organise the steps of a muscle biopsy processing in the correct order |  |  |
|  | Show the answer to above | 1. Personal protective equipment 2. Check patient details match form/specimen pot 3. Check and prepare equipment 4. Describe and measure muscle   5a. Cut muscle and triage to different samples  5b. Snap freeze muscle  5c. Place remainder of muscle in formalin  6. Dispose of sharps safely |  |
| Conclusion | Summarise steps |  |  |
