# Supplementary material for: How to Develop an Online Video for Teaching Health Procedural Skills: Tutorial for Health Educators New to Video Production
Source: JMIR Med Educ. 2024 Aug 7;10:e51740. doi: 10.2196/51740 (PMC11339575; doi:10.2196/51740)
Supplement: Multimedia Appendix 3 [file mededu_v10i1e51740_app3.docx]

**Title: Macroscopic processing of fresh skeletal muscle biopsy in a pathology laboratory**

Audience: Pathology registrars, consultants and histology technicians.

Learning outcomes:

1. State the equipment required for this procedure
2. Describe the steps of this procedure to a colleague
3. Describe how this process is different from formalin fixed tissue handling.
4. Identify the safety steps when performing this procedure.

| **Step** | **Images/Scenes** | **Audio/description (music/script)** | **Photos** |
| --- | --- | --- | --- |
| Title | Fresh skeletal muscle biopsy handling protocol in a Pathology laboratory | No music or narration required as title suffices | ADHB/and UoA logo  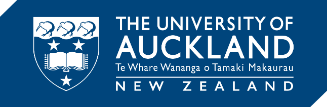  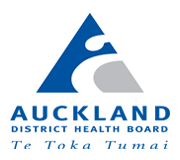 |
| Intro |  | Hi. I'm Komal Srinivasa, and I'm a consultant anatomical pathologist at Auckland City Hospital.  This is a video about how to handle fresh skeletal muscle tissue in a pathology laboratory.  In this demonstration, I'm using human tissue after consent from a patient.  But you may also practice the technique using a piece of red meat |  |
| Learning objectives | List of learning objectives (20s) |  | List of LO shown:   1. State the equipment required for this procedure 2. Describe the steps of this procedure to a colleague 3. Describe how this process is different from formalin fixed tissue handling. 4. Identify the safety steps when performing this procedure. |
| Step 1 | Personal protective equipment | Narration: First of all, put on your personal protective equipment.  This is an important safety step | Show animation of gown, gloves and  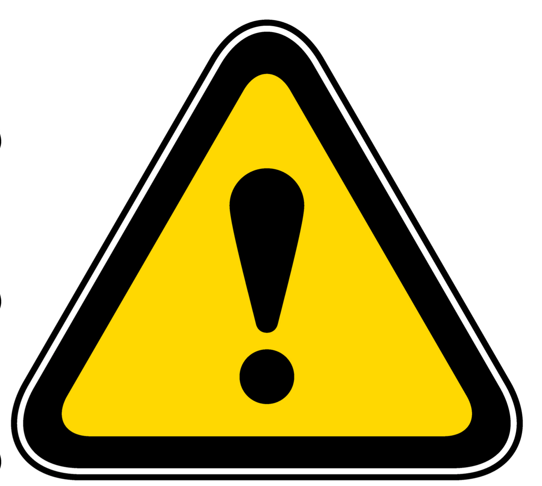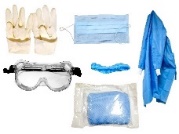goggles |
| Step 2 | Check patient details match form/specimen container | Narration: Check that the patient details match those on the form and specimen pot | Scene showing actor checking/looking at form whilst wearing PPE  (still shot) |
| Step 3 | Check and prepare equipment  List equipment with picture of each appearing matching the voice over  Scalp blade to cut, petri dish to put muscle on, ruler, medium for EM, containers for freezing, cork for freezing, liquid nitrogen, isopentane  Dissecting microscope, Thermometer, dental wax, ink | Narration: check and prepare the equipment.  Here is a list of the equipment required.  You may wish to pause the video to look at this in more detail. Describe and measure the muscle. | 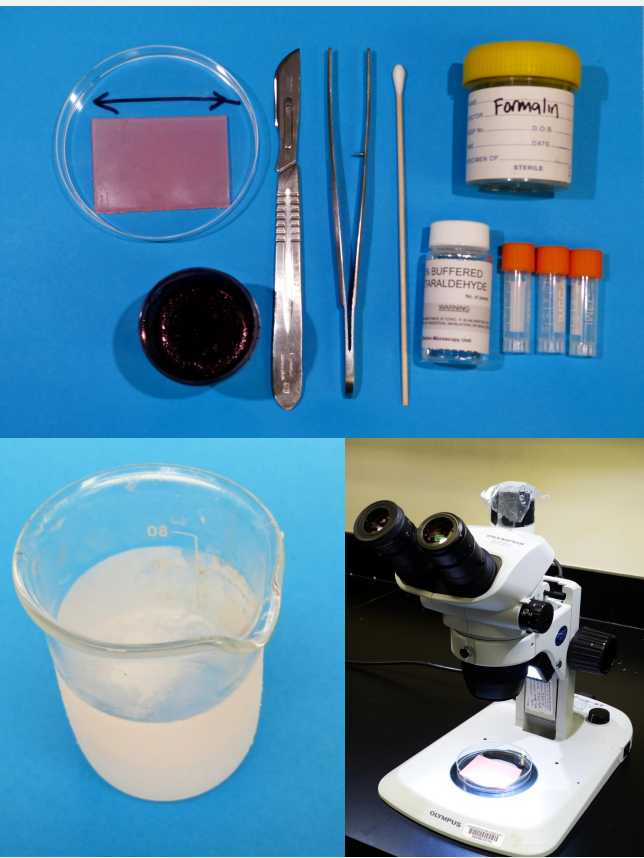line equipment up and can label later with animation |
| Step 4 | Describe and measure muscle | Narration: The specimen is a red piece. X by Y by Z millimetres in size. | Actor measuring muscle |
| Step 5 | Cut muscle and triage to different samples | Orientate the muscle cut and submit samples. |  |
| Step 5a | Snap frozen for histology | The cut muscle is placed in gum tragacanth moulding medium and snap frozen using liquid nitrogen (temp (<-130C). | Show actor cutting |
| Step 5b | Submit sample for electron microscopy | Submit sample for electron microscopy.  This is a longitudinal slice of muscle placed in glutaraldehyde fixation medium. |  |
| Step 5c | Submit samples for mitochondrial studies and DNA storage | submit samples for mitochondrial studies and DNA storage.  These are small pieces of muscle that are placed in centrifuge tubes or NUNC tubes and snap frozen.  The frozen samples will be stored in a -80 degrees Celsius freezer until required. |  |
| Step 5d | Place remainder of muscle in formalin for fixation | Mark the longitudinal and cross-section  portions of the muscle biopsy with coloured ink to allow easy orientation at the time of embedding. Transfer the pieces of muscle into formalin for fixation. |  |
| Step 6 | Dispose of sharps safely | Make sure to dispose of the scalpel blade safely. | On screen indicate important safety step  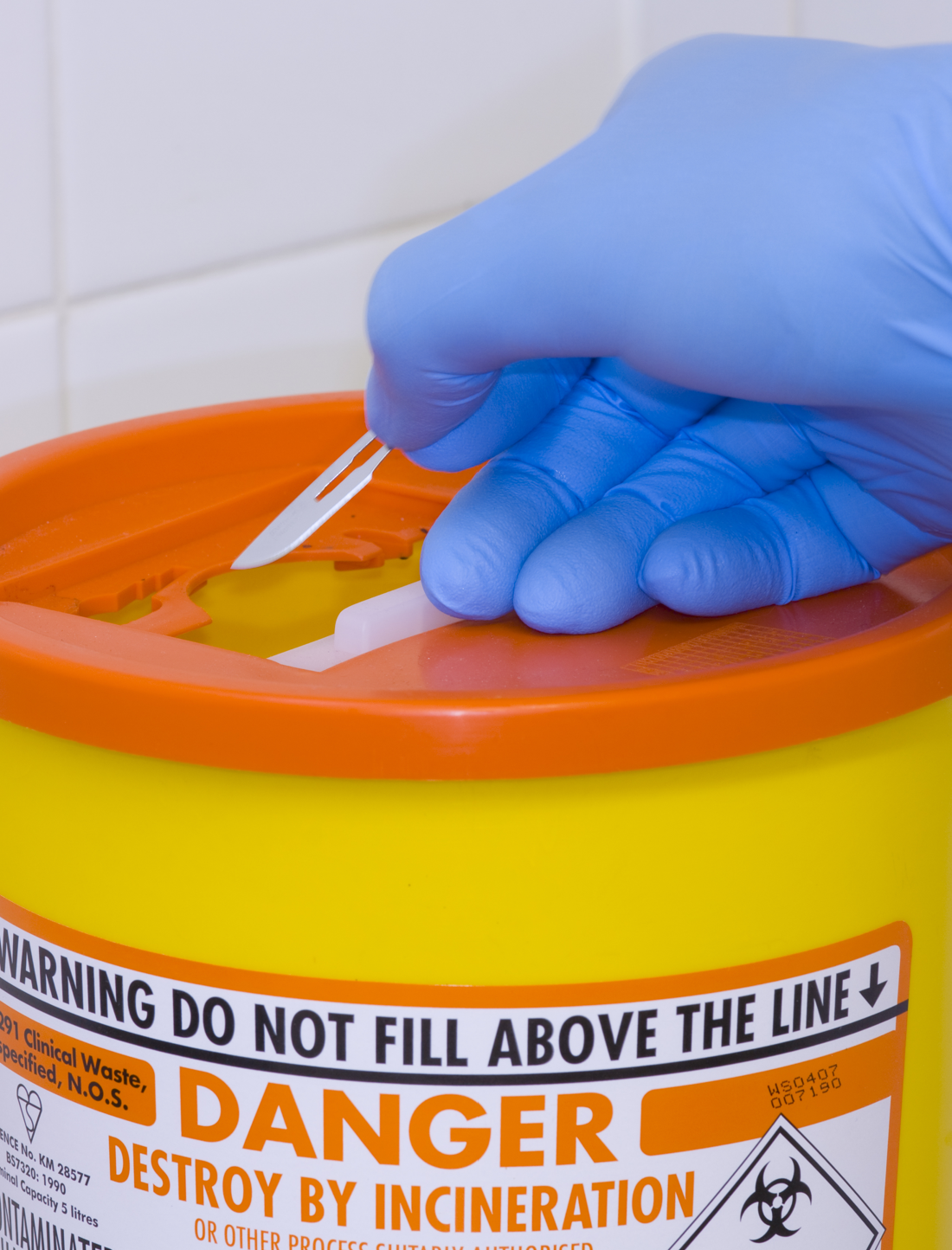 |
|  | Active learning:  Organise the steps of a muscle biopsy processing in the correct order | Now for active learning, drag and drop the tiles into the correct order of processing a muscle biopsy. |  |
